# Successful trabeculotomy in a patient with corticosteroid-induced glaucoma with anti-aquaporin 4 antibody-positive neuromyelitis optica: a case report

**DOI:** 10.1186/1752-1947-7-101

**Published:** 2013-04-10

**Authors:** Masahide Kawamura, Masahiro Zako

**Affiliations:** 1 Department of Ophthalmology, Aichi Medical University, Nagakute, Aichi, 480-1195, Japan

**Keywords:** Anti-aquaporin 4 antibody-positive, Corticosteroid-induced glaucoma, Neuromyelitis optica, Trabeculotomy

## Abstract

**Introduction:**

Corticosteroid therapy is a first-choice treatment for anti-aquaporin 4 antibody-positive neuromyelitis optica. Although we expected corticosteroid-induced glaucoma as a potential complication of the therapy, there are no reports in the literature describing it. In this report, we describe a case of successful trabeculotomy performed on a patient with corticosteroid-induced glaucoma and anti-aquaporin 4 antibody-positive neuromyelitis optica.

**Case presentation:**

A 40-year-old Japanese woman who was given prednisolone orally after the diagnosis of anti-aquaporin 4 antibody-positive neuromyelitis optica experienced acute, painful loss of vision in her right eye. Although her right eye intra-ocular pressure was increased, we considered the main cause of her recent visual disturbance to be neuromyelitis optica because her right eye visual acuity declined to no light perception within a short period with a marked central scotoma. We treated our patient with high-dose methylprednisolone and double-filtration plasmapheresis; however, no improvement was observed. After we performed trabeculotomy in her right eye, our patient’s post-operative intra-ocular pressure was maintained within the normal range. Her visual acuity drastically improved soon after the decrease of intra-ocular pressure.

**Conclusions:**

Both neuromyelitis optica and glaucoma caused our patient’s visual disturbance, and clinicians should plan for treatment of both neuromyelitis optica and glaucoma in such cases.

## Introduction

Intravenous and oral corticosteroid therapies are commonly used to treat anti-aquaporin 4 antibody-positive neuromyelitis optica (NMO), and plasmapheresis is also beneficial for patients with acute, severe vision loss who have optic neuritis that is refractory to corticosteroid therapy [1,2]. Because corticosteroid therapy is a first-choice therapy for anti-aquaporin 4 antibody-positive NMO, we should expect corticosteroid-induced glaucoma as a potential complication of the therapy. However there are no reports describing corticosteroid-induced glaucoma and its treatment in the context of a patient with anti-aquaporin 4 antibody-positive NMO. Here we describe a case of successful trabeculotomy performed on a patient with corticosteroid-induced glaucoma and anti-aquaporin 4 antibody-positive NMO.

## Case presentation

A 28-year-old Japanese woman first presented 13 years ago after experiencing acute, painful vision loss in her left eye with the appearance of a central scotoma as shown by Goldmann perimeter (GP); she was diagnosed as having retrobulbar optic neuritis, and treated with intravenous high-dose methylprednisolone (HDMP; 1000mg) followed by a tapering dose of oral prednisolone (PSL). Her visual loss slowly and incompletely recovered. Magnetic resonance imaging (MRI) analysis performed on her brain, orbit, and spine revealed a mild enhancement of her left optic nerve, cervical spinal cord, and thoracic spinal cord, with no other abnormalities. As a follow-up dose, low-dose PSL (5 to 10mg/day) was administered orally.

For 12 years after her initial visual problem, our patient’s best-corrected visual acuity (BCVA) has been 1.2 in her right eye and 0.15 in her left eye, and a left relative afferent pupillary defect has been shown. Three years ago, serum anti-aquaporin 4 antibody was observed. As our patient had optic neuritis, myelitis, MRI evidence of a contiguous spinal cord lesion of five segments in length and NMO-IgG seropositivity, she was diagnosed as having anti-aquaporin 4 antibody-positive NMO according to the diagnostic criteria for NMO [3]. Our patient experienced relapses of left retrobulbar optic neuritis 10 times during the 12 years after the initial episode. Every recurrence of left retrobulbar optic neuritis was treated with intravenous HDMP followed by a tapering dose of oral PSL. As a follow-up dose, low-dose PSL was always administered orally.

**Figure 1:**
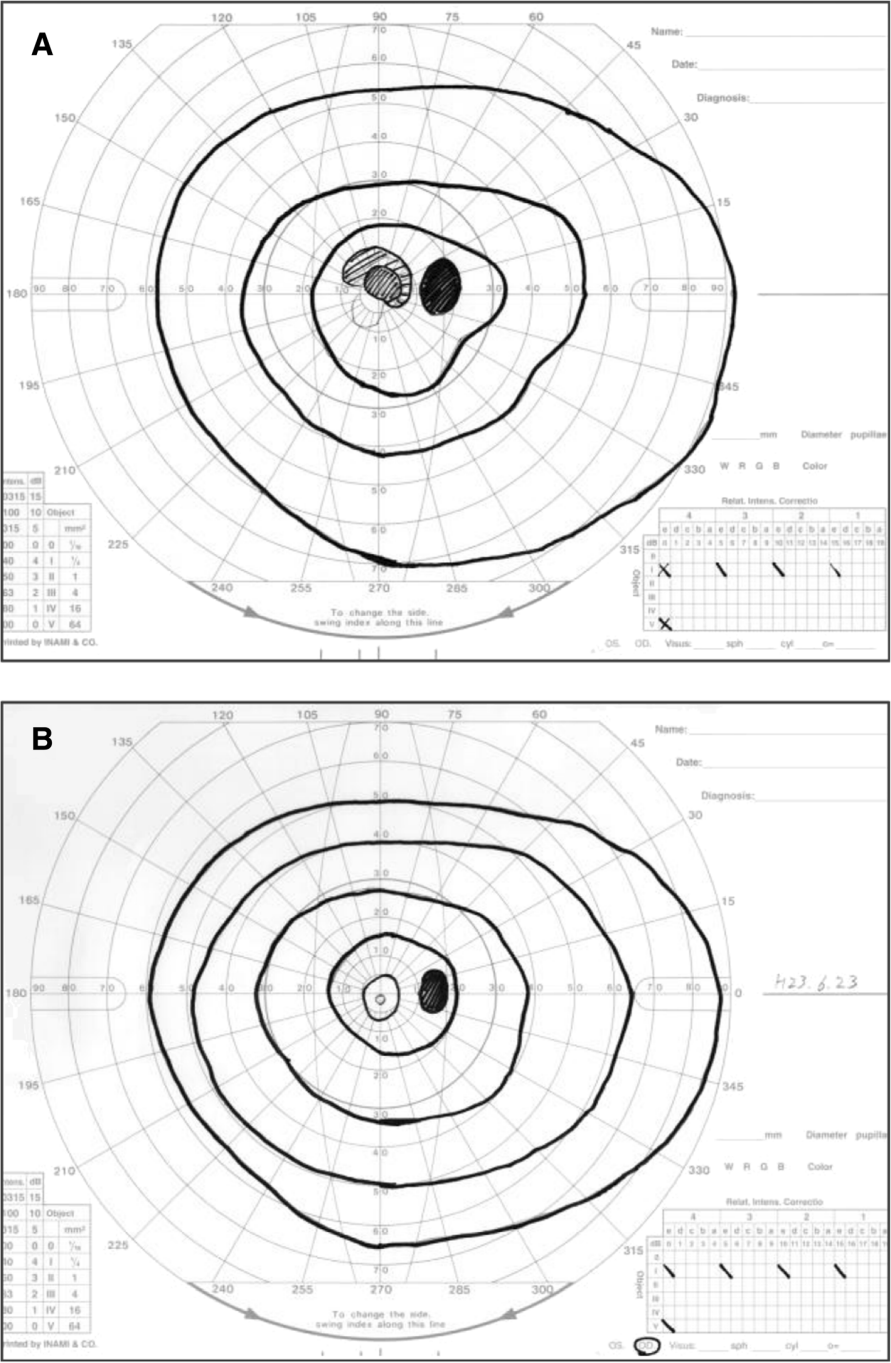
**The visual field of our patient’s right eye, analyzed by Goldmann perimeter. (A)** Central scotoma was detected in April 2011. **(B)** The central scotoma disappeared after treatment with intravenous high-dose methylprednisolone, double-filtration plasmapheresis, and trabeculotomy.

Last year, our patient presented with progressive vision loss in her right eye that had begun two days earlier. She also had mild pain with eye movement. Her right BCVA was 0.2 accompanied by reduced color perception, her GP test showed a central scotoma (Figure 1A), and both pupils exhibited poor responses to light stimulation. On funduscopic examination, her right optic disk appeared normal. An MRI analysis performed on her brain, orbit, and spine revealed no significant enhancement.

Our patient’s right intra-ocular pressure (IOP) had been 30 to 40mmHg, and she was also diagnosed as having corticosteroid-induced glaucoma in her right eye. She was treated with intravenous d-mannitol and acetazolamide followed by oral acetazolamide, oral potassium l-aspartate, topical dorzolamide hydrochloride, topical carteolol hydrochloride, and topical latanoprost. After these treatments, our patient’s right IOP was transiently reduced to 20 to 30mmHg.

**Figure 2:**
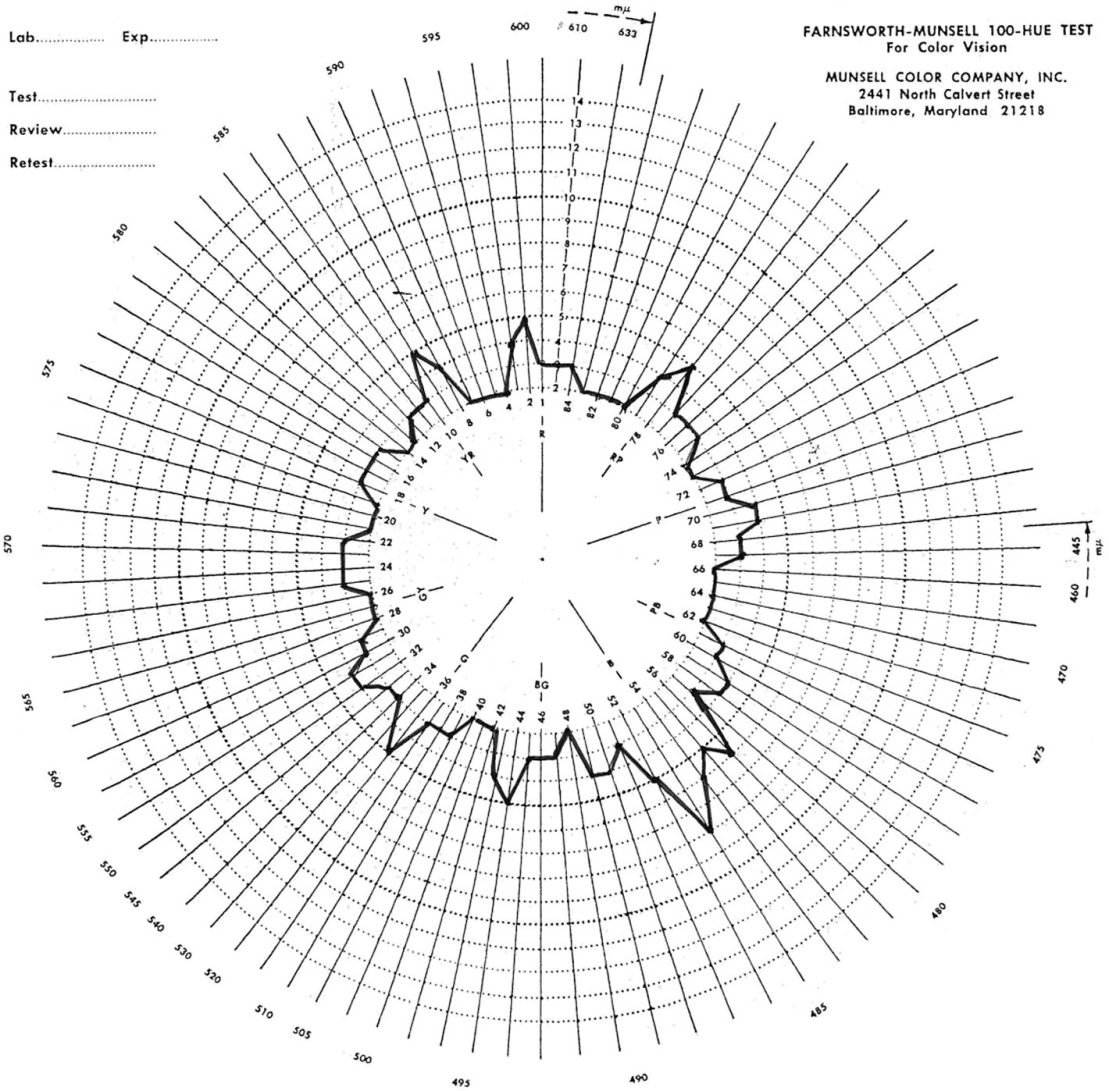
**A polar diagram of our patient’s right eye, examined using the Farnsworth-Munsell 100-hue color vision test after improvement over her last episode.** This test revealed a mild but non-specific pattern of defect. Our patient was unable to complete the test with her left eye because of the existing visual disturbance (not shown).

**Figure 3:**
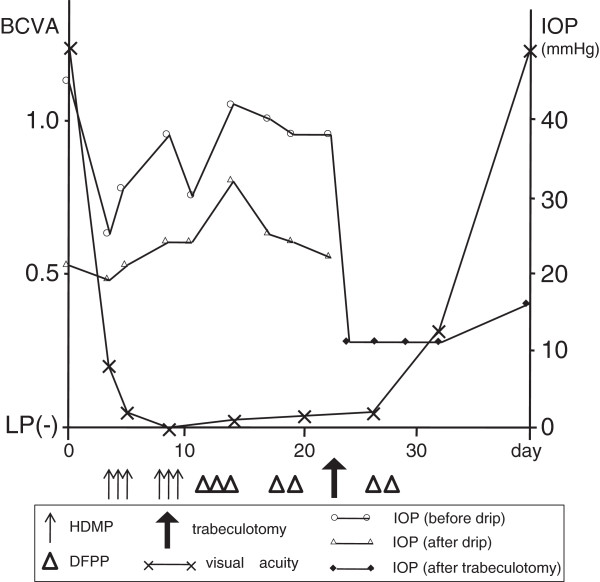
**A schema showing our patient’s right eye best-corrected visual acuity (BCVA), intra-ocular pressure (IOP), and treatments performed on our patient.** DFPP, double-filtration plasmapheresis; HDMP, high-dose methylprednisolone; LP(−), no light perception.

Because we considered anti-aquaporin 4 antibody-positive NMO to be the primary cause of our patient’s visual disturbance, we treated her with two courses of intravenous HDMP followed by a tapering dose of oral PSL. However, her right visual acuity declined to no light perception, and then we added double-filtration plasmapheresis (DFPP). After five consecutive days of DFPP, her visual acuity slowly improved. Because the increased IOP was undesirable for her right eye, we performed a trabeculotomy to reduce her right IOP; the post-operative IOP maintained under 15mmHg. After the trabeculotomy, we performed the remaining DFPP for two consecutive days. Two weeks later, our patient’s right BCVA recovered to 1.2, and this BCVA has now persisted for more than seven months. Final GP demonstrated no central scotoma (Figure 1B). The Farnsworth-Munsell 100-hue color vision test performed on her right eye after improvement over the last episode demonstrated a mild but non-specific pattern of consequent abnormality (Figure 2). She was unable to complete the test with her left eye because of the existing visual disturbance (data not shown). Changes in our patient’s right BCVA and IOP in the context of the performed treatments are summarized in Figure 3.

## Discussion

Intravenous and oral corticosteroid therapies are a first choice to treat anti-aquaporin 4 antibody-positive NMO [1,2]. However, corticosteroid-induced glaucoma is a possible complication of corticosteroid therapies, as shown here. In the present case, our patient’s right BCVA radically declined to no light perception in a short period of time and was accompanied by central scotoma, and therefore we initially considered the main cause of her visual disturbance to be anti-aquaporin 4 antibody-positive NMO rather than corticosteroid-induced glaucoma. We initially treated her with HDMP and DFPP; however, her BCVA drastically improved by relieving IOP through trabeculotomy. Based on the post-operatively consistent timing of decreased IOP and increased BCVA, we hypothesize that the trabeculotomy contributed remarkably to our patient’s visual improvement in this case. Although prior HDMP and DFPP might have been effective at achieving this timing by chance, it is impossible to verify it. This was a rare case in which the initial main cause of our patient’s visual disturbance was likely anti-aquaporin 4 antibody-positive NMO, but the critical treatment for her visual improvement appeared to be trabeculotomy against glaucoma. Axonal flow in the retina or retinal blood flow may have been considerably disturbed by the increased IOP, which might have further deteriorated visual function already damaged by anti-aquaporin 4 antibody-positive NMO, and this dysfunction might have been promptly improved by the decrease of IOP after HDMP and DFPP therapies. To the best of our knowledge, this is the first case in the literature to describe a successful course achieved by trabeculotomy on a patient with anti-aquaporin 4 antibody-positive NMO who experienced corticosteroid-induced glaucoma.

## Conclusions

Corticosteroid therapy is a first-choice treatment for anti-aquaporin 4 antibody-positive NMO, and corticosteroid-induced glaucoma is a potential complication of this therapy. Both NMO and glaucoma caused our patient’s visual disturbance, and clinicians should plan for the treatment of both NMO and glaucoma.

## Consent

Written informed consent was obtained from the patient for the publication of this case report and any accompanying images. A copy of the written consent is available for review by the Editor-in-Chief of this journal.

## Competing interests

The authors declare that they have no competing interests.

## Authors’ contributions

MK analyzed and interpreted the patient data. MZ was a major contributor in writing the manuscript. Both authors read and approved the final manuscript.
